# A Retrospective Analysis of Needle Thoracostomies at a Tertiary Level 2 Trauma Center

**DOI:** 10.7759/cureus.55736

**Published:** 2024-03-07

**Authors:** Sarthak Parikh, Maryavis Howell, Hung-Wen Yeh, Mani Cheruvu, Robert Goodwin, John Shellenberger

**Affiliations:** 1 Trauma Institute, Saint Francis Health System, Tulsa, USA; 2 Department of Orthopedic Surgery, Oklahoma State University, Tulsa, USA; 3 Center for Clinical Research and Sponsored Programs, Saint Francis Health System, Tulsa, USA; 4 Division of Health Services and Outcomes Research, University of Missouri-Kansas City School of Medicine, Kansas City, USA; 5 Division of Health Services and Outcomes Research, Children’s Mercy Kansas City, Kansas City, USA

**Keywords:** acute trauma care, pneumothorax (ptx), chest wall trauma, needle decompression, needle thoracostomy

## Abstract

Background: A tension pneumothorax is a condition that results in elevated pressure within the pleural space. The effective management of tension pneumothorax relies on needle decompression, commonly performed at the second intercostal space (ICS) midclavicular line (MCL). However, some literature suggests that catheters placed in the second intercostal space midclavicular line are prone to higher failure rates compared to the fifth intercostal space midaxillary line (MAL) (42.5% versus 16.7%, respectively). In this study, we aim to identify and scrutinize the prevalence of prehospital needle decompression from one tertiary care center over eight years and examine their trends, efficacies, or pitfalls. It is hypothesized that preclinical providers are performing needle decompression prematurely and unnecessarily.

Methods: A set of 90 patient records obtained using the trauma registry at Saint Francis Hospital, Tulsa, Oklahoma, were retrospectively reviewed to evaluate the management and outcomes of tension pneumothorax, as well as the indications documented for needle decompression. Patient charts were reviewed via Epic Hyperspace (Epic, Madison, WI). The Oklahoma Emergency Medical Service Information System (OKEMSIS) also provided information contributing to the sample population.

Results: The most documented indications for needle decompressions included diminished or absent breath sounds (52.70%), hypoxia (15.54%), hypotension, and hemodynamic instability (6.76%). Emergency medical services (EMS) reported improvements in 51 (56.67%) patients after needle thoracostomy. Improvements in vital signs after needle decompression were sporadic. The most common complication was catheter dislodging, which occurred most in the second intercostal space midclavicular line. Only nine patients had an oxygen saturation (SpO_2_) below 92% and a systolic blood pressure (SBP) below 100 mm Hg prior to receiving needle decompression.

Conclusion: Current practices for tension pneumothorax show little improvement in vital signs before and after needle decompression. Vital signs prior to needle decompression often do not indicate tension pneumothorax physiology. Preclinical providers may be inappropriately performing needle decompressions, an invasive procedure with complications.

## Introduction

The needle decompression of the thoracic cavity is a common procedure performed in prehospital settings as an immediate response to tension pneumothorax [1]. Tension pneumothorax is a severe condition that results in elevated pressure within the pleural space, leading to lung compression, which results in mediastinal shift, decreased venous return, hypoxia, and eventual cardiovascular collapse [1,2]. The condition may present in prehospital contexts, often as a consequence of trauma [1,2]. Common prehospital interventions, such as positive-pressure ventilation, can also rupture the visceral pleura and cause an iatrogenic tension pneumothorax [3]. The manifestations of tension pneumothorax often involve difficulty breathing, chest pain, rapid heartbeat, tracheal deviation, jugular venous distension, reduced breath sounds, and eventual hemodynamic collapse [1]. Clinical diagnosis may be problematic as the degree of breathlessness may not align with the pneumothorax size and an impending tension pneumothorax may be difficult to distinguish from a simple pneumothorax [1,4]. Moreover, early intervention prevents the development of late diagnostic findings, which further convolutes a definitive diagnosis. Hence, the diagnostic proficiency of preclinical clinicians is challenged when discerning the presence or absence of tension pneumothorax in a patient while managing complex trauma patients in the prehospital setting. This is particularly true given that experienced and well-trained providers are more likely to perform needle decompressions accurately [5,6].

Even though the occurrence of tension pneumothorax among prehospital trauma patients is relatively rare (ranging from 0.2% to 1.7%), the mortality rate for those left untreated is high [7,8]. Nevertheless, the efficacy and relevance of needle decompressions have been scrutinized due to adverse events and failure rates. The possible complications include mechanical obstruction of the catheter, lung contusion, pneumonia, empyema, and life-threatening iatrogenic injury to major visceral organs or vascular structures [9-11]. The complication rates of needle decompression range from 2% to 25%, and the failure rate can reach as high as 76% depending on the study, population, thoracostomy location, and provider experience [7,12]. Therefore, effective diagnosis and treatment are imperative to optimize patient outcomes in the preclinical setting.

The definitive management of tension pneumothorax relies heavily on the decompression of the thoracic cavity [13]. The swift decompression of the pleural space alleviates the pressure by allowing air to escape and enabling lung inflation and function [13]. In the prehospital setting, the decompression of a presumed tension pneumothorax is done by needle decompression. This is usually replaced with a formal chest tube after arrival at the hospital if pneumothorax persists [14]. Needle thoracostomy is performed in one of three common locations: the second intercostal space (ICS) at the midclavicular line (MCL), the fourth or fifth ICS anterior axillary line (AAL), or the fifth ICS midaxillary line (MAL) [13]. The second ICS MCL is the most common decompression location. More recent literature suggests that catheters placed in the second ICS MCL are prone to higher failure rates compared to the fifth ICS MAL (42.5% versus 16.7%, respectively) [15,16]. Ultimately, chest thoracostomies are the definitive treatment for tension pneumothorax [3].

Discrepancies in the location used to perform needle decompression illustrate the varied practice patterns in the prehospital setting, necessitating a standardized and effective needle decompression protocol that ensures successful prehospital tension pneumothorax diagnosis and treatment with limited adverse effects. The current literature suggests an ongoing debate regarding the effectiveness and success of prehospital needle decompression [5,13,17-19]. It is hypothesized that preclinical providers are performing needle decompression prematurely and unnecessarily. In this study, we aimed to identify and scrutinize the prevalence of prehospital needle decompression from one tertiary care center over eight years and examine their trends, efficacies, and pitfalls.

## Materials and methods

This observational retrospective chart review study was approved by the Saint Francis Health System Institutional Research Ethics Board (IREB number: 2324-23) and determined not to qualify as human research. As a result, this project does not require oversight by the IREB. Patient consent was not required as contacting the patient would require obtaining information that would otherwise be unnecessarily accessed and risk patient privacy. The charts of the patients who underwent needle decompression in the prehospital setting prior to entering a 1,112-bed tertiary care center and a state level 2 trauma center were reviewed. The patients or the public were not involved in the design, conduct, reporting, or dissemination plans of our research as this would add bias to the study. Using the Digital Innovations Version 5 (DIv5) Trauma Registry, a query search was performed from 1/1/2015 to 7/5/2023 to identify all patients meeting the inclusion criteria for entry in the state of Oklahoma and the inclusion criteria for entry in the National Trauma Data Standard. This query included 29,294 patients with the following data: trauma number, date of arrival, medical record number, name, inclusion source, prehospital mode, arrival at destination time, and prehospital interventions performed. This data was cross-referenced with information for all transfers to the hospital requiring needle decompression between 2018 and 2022, which was also provided by the state’s Department of Health.

A final list of 90 patients was identified by the cross-referencing process. The data was collected via chart review on Epic Hyperspace (Epic, Madison, WI) and the Oklahoma Emergency Medical Service Information System (OKEMSIS). The following data from Epic and OKEMSIS was identified, extracted, compared, and analyzed: height, weight, body mass index (BMI), sex, age, trauma type, the mode of transport, needle locations, the quantity of needle decompressions performed, indications for emergency medical service (EMS) needle decompression, needle size, initial chest X-ray, initial computed tomography (CT) scan, the presence of pneumothorax on imaging, the presence of rib fractures on imaging, pre-needle decompression vitals, post-needle decompression vitals, proper needle thoracostomy placement, chest tube placement and indication, iatrogenic injury, alive or deceased status, EMS-reported improvement, and complications. Emergency medical service-reported improvements included any narrative documentation specifically stating that the patient’s overall status, vital signs, or breathing improved after the needle decompression. Microsoft Excel 2010 (Microsoft Corp., Redmond, WA) was used to organize and analyze the final set of 90 patients because it was the software present on the secure hospital computers. Once the data was extracted, patient identifiers were removed and replaced with generic numbers (patient 1). Information was then analyzed to identify indications for needle thoracostomy, changes in vital signs, and patients with hemodynamic instability necessitating the needle thoracostomy intervention.

Frequencies and percentages were used to summarize categorical outcomes. Continuous variables were summarized by their median and interquartile range (IQR), and missing values were imputed by Multiple Imputation by Chained Equations using the predictive mean matching method 10 times [19]. The pre- and post-needle decompression changes in systolic blood pressure (SBP), diastolic blood pressure (DBP), heart rate (HR), oxygen saturation (SpO_2_), and respiratory rate (RR) were then compared using the Wilcoxon signed-rank test due to skewed distributions and/or outliers, and the results were pooled by Rubin’s rule [20].

## Results

A total of 90 patients who underwent prehospital needle decompression were identified over eight years (Table 1). Among them, 16 were female (17.78%), and 71 were male (78.89%). For three patients, their sex was not identified (3.33%). Fifty-four (60.00%) patients were transported to the hospital by ground transport, 33 patients (36.67%) arrived by air transport, and transport information was not identified for three patients (3.33%). The average BMI for the sample was 26.82 kg/m^2^. The average age for the sample was 44 years. Accidents involving motor vehicles were the most common presentation (47 cases, 52.22%), and penetrating trauma was the second most common (27 cases, 30.00%). The majority of the patients remained alive through the hospital course (61 patients, 67.67%).

**Table 1:** Patient Demographics BMI: body mass index

Demographic	n	%
Sample size	90	100%
Sex		
Male	71	78.89%
Female	16	17.78%
Unidentified	3	3.33%
Transport		
Ground	54	60.00%
Air	33	36.67%
Unspecified	3	3.33%
BMI	26.82	-
Age	44	-
Trauma types		
Motor vehicle accident	47	52.22%
Drug overdose	1	1.11%
Penetrating trauma	27	30.00%
Others	5	5.56%
Alive or deceased		
Alive	61	67.78%
Deceased	19	21.11%
No data	10	11.11%

A total of 117 needle thoracostomies were performed, and 86 were documented in the second ICS MCL (63, 53.85%), fifth ICS AAL (seven, 5.98%), and fifth ICS MAL (16, 13.68%). Eighteen patients (20%) had more than one needle thoracostomy. Thirty-one needle thoracostomies (26.50%) did not have a documented location or decisive location information on imaging. A chest tube was placed in 57 of the 90 patients who received needle decompression (63.33%). Pneumothorax (37, 64.91%), hemothorax (eight, 14.04%), and hemopneumothorax (five, 8.77%) were the most common indications for chest tube placement. Seven patients (12.28%) received a chest tube with no specific indication documented.

There were 148 indications and combinations of indications for needle decompression documented by EMS. Table 2 ranks these indications from 1 to 10, 1 being the most reported and 10 being the least reported. Diminished or absent breath sounds (78 patients, 52.70%) were the most documented, followed by hypoxia (23 patients, 5.54%) and hypotension and hemodynamic instability (10 patients, 6.76%). More specific indicators of tension pneumothorax, such as jugular venous distension (two patients, 1.35%) and tracheal deviation (seven patients, 4.73%), were mentioned less frequently. Other documented indications included imaging, unequal respirations, cyanosis, diminished pulses, chest trauma, cardiac arrest, hyperinflation, difficulty ventilating, and subcutaneous air (six patients, 4.05%). Ten patients did not have specific indications recorded in their OKEMSIS documentation (6.76%).

**Table 2:** Most Common Indications for Prehospital Needle Decompression

1 (most common) to 10 (least common)	Ranking of indications mentioned
1	Diminished/absent breath sounds
2	Hypoxia
3	Hypotension/hemodynamic instability
4	Did not specify
5	Tracheal deviation
6	Unequal respirations
7	Other indications: diminished pulses, chest trauma, cardiac arrest, hyperinflation, difficulty ventilating, and subcutaneous air
8	Imaging or confirmation by a physician
9	Jugular venous distension
10	Cyanosis

Chest X-rays and CT scans conducted at the time of admission were analyzed to identify cases of pneumothorax or rib fractures. Of the 90 patients in the cohort, 49 (54.44%) had pneumothorax, 31 (34.44%) had rib fractures, and 23 (25.56%) had both on imaging at the time of admission. Nineteen patients (21.11%) did not have radiographs to diagnose the presence of pneumothorax on presentation. Twenty patients (22.22%) did not have radiographs to diagnose the presence of rib fractures on presentation.

EMS showed improvements in 51 (56.67%) patients after needle thoracostomy. Twenty-three patients (25.56%) demonstrated no improvement. One patient reported a worsening condition (4.35%) after decompression, and 16 patients (17.78%) had neither improvement nor worsening conditions recorded in their OKEMSIS documentation. Eighteen patients (20.00%) of the 90 suffered complications, including iatrogenic pneumothorax (one patient, 5.56%), improper placement/dislodged needle (12 patients, 66.67%), broken needle (two patients, 11.11%), traumatic pneumatocele (two patients, 11.11%), and biliary drainage (one patient, 5.56%). Iatrogenic pneumothorax was defined as documentation explicitly stating that an iatrogenic pneumothorax was present. Nine complications occurred in the second ICS MCL. One complication (improper placement/dislodging/poor penetration) was documented in the fifth ICS AAL, and three complications (improper placement/dislodging/poor penetration) were documented in the fifth ICS MAL. Five complications occurred in unspecified locations. This data was documented in the patient chart by physicians or in the OKEMSIS report by the EMS crew. Improper placement or needle dislodgement was identified by analyzing radiographs and/or explicitly documented in the physician notes.

Vital signs before and after needle decompression were documented in the OKEMSIS reports for 64 patients undergoing a first needle decompression and 15 patients undergoing a second needle decompression. The average SBP, DBP, HR, RR, and SpO_2_ before the first needle decompression were 112.12 mm Hg, 68.77 mm Hg, 102.09 beats per minute, 27.02 breaths per minute, and 89.14%, respectively. The median vital sign values and proportions of the observed vital signs are reported in Table 3. SpO_2_ and HR were the only values that demonstrated a statistically significant change after the first and second needle decompressions, respectively.

**Table 3:** Median Vital Signs After Needle Decompression §Interquartile range (IQR) †Wilcoxon signed-rank test on the pooled estimates from 10 imputed datasets ‡P-value adjusted by the Benjamini-Hochberg procedure to control false discovery rate (FDR) at the 0.05 level

Vital Sign	Before		After		P-value	
-	Number of observations (%)	Median (IQR§)	Number of observations (%)	Median (IQR§)	Raw†	FDR adjusted‡
Systolic blood pressure (mm Hg)	44 (69%)	110 (82-136)	52 (81%)	118 (101-139)	0.045	0.075
Diastolic blood pressure (mm Hg)	44 (69%)	68 (53-84)	51 (80%)	72 (64-85)	0.022	0.054
Heart rate (beats per minute)	56 (88%)	103 (85-118)	59 (92%)	100 (90-112)	0.257	0.257
Oxygen saturation (SpO_2_)	45 (70%)	92 (84-95)	47 (73%)	95 (90-98)	0.001	0.006
Respiratory rate (breaths per minute)	48 (75%)	24 (17-30)	54 (84%)	20 (16-28)	0.229	0.257

A retrospective diagnosis of tension pneumothorax physiology was evaluated by assigning a correct diagnosis with SpO_2_ < 92% and SBP < 100 mm Hg. Of the 44 patients who had a documented pre-needle decompression SpO_2_ value, 18 had SpO_2_ of below 92%. Of the 44 patients who had a documented pre-needle decompression SBP, only nine patients had SpO_2_ < 92% and SBP < 100 mm Hg (Table 4). Of the patients who underwent a second needle decompression, only one patient had SpO_2_ < 92% and SBP < 100 mm Hg.

**Table 4:** Patients With Tension Pneumothorax Defined as Oxygen Saturation (SpO2) < 92% and Systolic Blood Pressure (SBP) < 100 mm Hg After Needle Decompression

Diagnosis of tension pneumothorax by assigning a correct diagnosis with SpO_2_ < 92% and SBP < 100 mm Hg	
Number of patients with a documented pre-needle decompression SpO_2_	44
Number of patients with a pre-needle decompression SpO_2_ < 92%	18
Number of patients with a documented pre-needle decompression SBP	43
Number of patients with pre-needle decompression SpO_2_ < 92% and SBP < 100 mm Hg	9

## Discussion

This study was a retrospective review of patients who underwent prehospital needle thoracostomy for a presumed tension pneumothorax. We intended to evaluate the indications and efficacy of needle decompressions. Many of the patients in this cohort received a needle thoracostomy before developing tension pneumothorax, which conceals the true incidence given that tension pneumothorax is a clinical diagnosis based on signs that appear late. EMS providers in this study predominantly diagnosed impending tension pneumothorax based on the absence of breath sounds and hypoxia in thoracic trauma patients, likely to prevent the exacerbation of hemodynamic compromise. Hemodynamic instability was rarely present, and improvement in vital signs was sporadic. Complications were prevalent, and immediate improvements occurred in only 56.67% of the cases. The majority of complications included improper placement and poor penetration. Poor penetration was most common in the second ICS MCL. Lastly, of the 90 patients, 37 patients received a chest tube for pneumothoraces, and 13 patients received a chest tube for hemothoraces or hemopneumothoraces. The presence of hemothoraces in patients undergoing needle decompression for tension pneumothorax may reveal difficulties for prehospital clinicians in distinguishing between hemothorax and pneumothorax diagnosis. Hypoxia and absent breath sounds are also present in hemothoraces. This may lead to more needle decompressions than necessary in the prehospital setting and emphasizes the need for improved education.

This data illustrates several discrepancies between the academic teachings of tension pneumothorax management and its clinical application. The indication for needle decompression is a tension pneumothorax, which causes mediastinal shift, decreased venous return, decreased cardiac output, and ultimately cardiovascular collapse [4]. As per the 2023 Medical Control Board Treatment Protocol of Oklahoma City and Tulsa, needle decompression is indicated for tension pneumothorax, which includes increasing respiratory insufficiency in a susceptible patient, SBP below 100 mm Hg, and three or more of the following: air hunger, cyanosis, decreased breath sounds on the affected side, jugular venous distension, and tracheal shift to the opposite side. Increased respiratory insufficiency applies to patients with spontaneous pneumothorax, CPR with pulseless electrical activity, difficulty bagging, an unresponsive sucking chest wound, and chest trauma with a suspected pneumothorax [21]. The cohort of patients in this study suffered from accidents related to motor vehicle collisions, gunshot wounds, stab wounds, or other mechanisms of chest trauma. However, only nine patients demonstrated SpO_2_ < 92% and SBP < 100 mm Hg immediately prior to the first needle decompression. These inconsistencies reveal a gap between the academic and clinical scenarios EMS providers face and raise the question of how a proper diagnosis can be achieved without delaying care. Needle decompression is the treatment for tension pneumothorax. Therefore, impending tension physiology should be present prior to decompressing the thorax, rather than solely relying on the absence of breath sounds. It is imperative that EMS training emphasizes skillful clinical diagnosis and management to reduce the rate of false-positive diagnoses and complications when performing needle decompression. Improving the accuracy of diagnosis will reduce the gap between the prevalence of needle decompressions and the prevalence of tension pneumothoraces.

One way to improve the outcomes of needle decompression is to identify the optimal location for catheter placement. The Advanced Trauma Life Support 10th Edition program, published in 2018, suggests improved success in reaching the thoracic cavity in cadaveric studies when using the fourth or fifth ICS MAL compared to the second ICS MCL [22,23]. A study conducted by Inaba et al. in 2012 found that needle thoracostomy failed in 42.5% of the cases at the second ICS MCL compared to 16.7% of the cases in the AAL [15]. This study also showed that needle thoracostomy placement was 100% successful in the fifth ICS and only 58% successful in the second ICS [15]. This may explain the high rates of poor penetration and the lack of improvement in our cohort. The current guidelines published by International Trauma Life Support in 2017 advocate for the use of simple thoracostomy, or finger thoracostomy, over needle thoracostomy due to its associated complications, including easy dislodging, inability to reach the pleural space, and iatrogenic injury to organs such as the heart, liver, spleen, or lungs. Simple thoracostomy performed over the fourth ICS MAL has been found to be clinically accurate, effective, and superior to needle decompressions. When compared with needle decompression, fewer patients undergoing simple thoracotomy were pronounced dead, and no cases of lung damage were associated with prehospital thoracostomy [20]. Although these guidelines are standard in some air medical transport services, they can be implemented in ground transport services to improve overall patient outcomes. The differences between common guidelines at the different state and international levels are apparent in Table 5. Finger thoracostomy can allow preclinical providers to readily treat a possible tension pneumothorax while avoiding the complications associated with needle thoracostomy.

**Table 5:** Education Guidelines and Recommendations From Different Trauma Services

Source	Recommendations
International Trauma Life Support (ITLS) [21]	Indication: the presence of tension pneumothorax with decompensation as evidenced by any of the following: respiratory distress and cyanosis, signs of shock, and decreasing level of consciousness
		The standard treatment for tension pneumothorax is needle decompression. However, ITLS urges providers to consider performing a simple thoracostomy, also known as a finger thoracostomy, instead of needle decompression due to their high failure rates, short catheters, kinking, and improper location
	Location: fifth intercostal space midaxillary line	
	EMS System for Metropolitan Oklahoma City and Tulsa 2023 Medical Control Board Treatment Protocols [22]	Indication: suspected tension pneumothorax
			Clinical signs: suspected pneumothorax AND systolic blood pressure of less than 100 mm Hg (<70 + (2 × age in years) mm Hg in pediatric patients) AND three or more of the following: air hunger, cyanosis, decreased breath sounds, jugular venous distension, and tracheal deviation
Location: second intercostal space midclavicular line			
		Contraindications: no absolute contraindications
Complications: tension pneumothorax, lung laceration, hemothorax, and infection		
		Avoid needle thoracostomy medial to the midclavicular line. Inferior to a rib and inferior to the right sixth intercostal space to avoid the upper margin of the liver
Maryland Institute for Emergency Medical Services Systems [23]		The site for needle thoracostomy in pediatric patients under 15 years of age is the second intercostal space midclavicular line
			Patients 15 years and older: fifth intercostal space at the anterior axillary line (preferred) or second intercostal space at the midclavicular line
		Indications: life-threatening tension pneumothorax in the extremis with absent lung sounds AND a clear evidence of hemodynamic compromise to include hypotension (systolic blood pressure < 100 mm Hg) and/or arrest

There are several limitations in this study. Although 90 patients were included, 25% of the patients did not have the same comprehensive information required for high statistical power. For instance, some patients lacked vital signs, and others lacked specifically mentioned decompression locations. In turn, formulating decisive conclusions can be difficult. Moreover, documentation was erratic, and the extrapolation of specific details, such as thoracostomy indications, was based partly on documentation and on interpretation. Catheters were often removed immediately after hospital admission, so imaging demonstrating proper insertion was based on physician documentation in most cases. Only a few cases of improper placement were assessed using imaging. Furthermore, variations in preclinical provider skillset and experience can introduce confounding factors that can affect the accuracy of needle placement and complication rates. These limitations were mostly due to the retrospective nature of this study and cannot establish causality or directly assess the efficacy of educational interventions or alternative management techniques. Future studies utilizing a prospective design with better control of variables are necessary to accurately capture needle decompression performance and outcomes. Future studies should also focus on BMI or obesity, the incidence of decompression failure/fallout in all three common placement locations, and outcomes following simple thoracostomy.

Ultimately, this was an observational study that aimed to analyze the prevalence of needle decompression and its outcomes from the perspective of a tertiary care center. The results of this study support that in the preclinical setting, needle decompressions may have been administered prematurely in the absence of tension physiology without improvement in vital signs. Nevertheless, the accurate diagnosis of tension pneumothorax is difficult in the prehospital setting because polytrauma patients may not present specific signs until decompensation. Improved EMS education and the standardization of tension pneumothorax diagnosis and treatment can help preclinical providers accurately identify impending tension pneumothorax and improve the ratio of decompressions to complications.

## Conclusions

Current practices for tension pneumothorax show little improvement in vital signs before and after needle decompression. Diminished or absent breath sounds are the most common indication for needle thoracostomy in the field. Vital signs prior to needle decompression often do not indicate tension pneumothorax physiology. Therefore, preclinical providers may be inappropriately performing needle decompressions. These procedures pose a risk to patients if performed improperly or without indications. Further education on proper tension pneumothorax diagnosis and management may help reduce complications and increase accuracy.
